# Immune Polarization Potential of the *S. aureus* Virulence Factors SplB and GlpQ and Modulation by Adjuvants

**DOI:** 10.3389/fimmu.2021.642802

**Published:** 2021-04-15

**Authors:** Daniel M. Mrochen, Patricia Trübe, Ilka Jorde, Grazyna Domanska, Cindy van den Brandt, Barbara M. Bröker

**Affiliations:** ^1^ Department of Immunology, University Medicine Greifswald, Greifswald, Germany; ^2^ Department of Medicine A, University Medicine Greifswald, Greifswald, Germany

**Keywords:** *Staphylococcus aureus*, vaccine, adjuvants, SplB, GlpQ, immune polarization, mouse models, Th2

## Abstract

Protection against *Staphylococcus aureus* is determined by the polarization of the anti-bacterial immune effector mechanisms. Virulence factors of *S. aureus* can modulate these and induce differently polarized immune responses in a single individual. We proposed that this may be due to intrinsic properties of the bacterial proteins. To test this idea, we selected two virulence factors, the serine protease-like protein B (SplB) and the glycerophosphoryl diester phosphodiesterase (GlpQ). In humans naturally exposed to *S. aureus*, SplB induces a type 2-biased adaptive immune response, whereas GlpQ elicits type 1/type 3 immunity. We injected the recombinant bacterial antigens into the peritoneum of *S. aureus*-naïve C57BL/6N mice and analyzed the immune response. This was skewed by SplB toward a Th2 profile including specific IgE, whereas GlpQ was weakly immunogenic. To elucidate the influence of adjuvants on the proteins’ polarization potential, we studied Montanide ISA 71 VG and Imject™Alum, which promote a Th1 and Th2 response, respectively. Alum strongly increased antibody production to the Th2-polarizing protein SplB, but did not affect the response to GlpQ. Montanide enhanced the antibody production to both *S. aureus* virulence factors. Montanide also augmented the inflammation in general, whereas Alum had little effect on the cellular immune response. The adjuvants did not override the polarization potential of the *S. aureus* proteins on the adaptive immune response.

## Introduction

*Staphylococcus aureus* (*S. aureus*) is both a human commensal and major pathogen that can cause a variety of diseases, ranging from skin and soft tissue infections like folliculitis, to life-threatening diseases such as sepsis or infective endocarditis (1). Twenty to 30% of individuals are persistently and asymptomatically colonized with *S. aureus*, while the others are intermittent carriers, i.e. phases of colonization alternate with phases of non-colonization (2–4). *S. aureus* belongs to the so-called ESKAPE bacteria, nosocomial pathogens that show high virulence and often multidrug resistance (5–7). The ESKAPE bacteria cause high mortality and large economic losses worldwide (8–11).

There is no effective vaccine against invasive *S. aureus* infections; so far all vaccine candidates have failed in clinical trials (12, 13). The reasons for this are manifold. On the one hand, *S. aureus* produces numerous, partly redundant virulence factors, many of which contribute directly to immune evasion. The pathogen also shows a high degree of adaptability due to its genomic plasticity (14, 15). On the other hand, the correlates of protection against *S. aureus* infection are not yet well understood. A high titer of *S. aureus*-specific antibodies is associated with protection against blood stream infection; nevertheless, all vaccines that were based on antibody production alone have failed (16, 17). A growing body of research highlights the importance of T cell mediated immunity against *S. aureus* infections. For instance, mouse models have shown that IL-17-producing T cells are crucial in resolving *S. aureus* skin infections, while IFNγ-producing T cells are critical during bloodstream infections (16, 18–20). In human *S. aureus* bacteremia patients, Greenberg *et al.* found that a higher Th17/Th1 cytokine response ratio was associated with increased mortality (21). Analysis of serum cytokines in individuals who developed an *S. aureus* infection despite vaccination with the vaccine candidate IsdB suggests that absent or misdirected T-cell responses can be fatal to disease outcome (22). These studies suggest that the quality or polarization of the immune response matters for protection against *S. aureus* and that distinctive T cell responses are needed for its control in different disease settings.

Several factors influence the profile of an immune response. The local environment of the confrontation with the pathogen can be decisive. In the lungs, for instance, Th2 cell responses are promoted (23). Pathogen-associated molecular patterns (PAMPs) affect how innate immune cells instruct the differentiation of adaptive immune cells (24–26). But also the antigens themselves, even single epitopes, can have polarizing potential, as it has been shown for various organisms (27–31). This is also true for the ubiquitous bacterium *S. aureus*, for which most humans have established an immune memory (4, 32–34). Different *S. aureus* antigens elicit different immune polarization profiles in a single person (35–37). This pre-existing immune polarization likely influences the reaction profile to further encounters with *S. aureus* antigens in infection or vaccination (18, 38, 39). In vaccination adjuvants are used to direct the response to a desired immune profile and to increase its intensity (40).

To study the intrinsic polarization potential of *S. aureus* antigens, we selected two virulence factors that are released by *S. aureus*: The serine protease-like protein B (SplB) is typically associated with a Th2 response (36), and the glycerophosphoryl diester phosphodiesterase (GlpQ) is described as a Th1/Th17-driving antigen (35). We prepared recombinant antigens, injected these – without adjuvant – into the peritoneum of *S. aureus*-naïve C57BL/6N mice and evaluated the quality of the immune reaction. To analyze the modulating effects of adjuvants, we applied the same antigens formulated with either the Th2-promoting adjuvant Imject™Alum (Alum) or the Th1-promoting adjuvant Montanide ISA 71 VG (Montanide) (41–43).

## Materials and Methods

### Recombinant Protein Production

Recombinant, C-terminal Strep-tagged SplB and GlpQ for the immunization of mice were produced in *E.coli* BL21 (DE3) pLysS. Cells were lysed using a sonicator and debris removed by centrifugation. The proteins were purified from lysate by the means of affinity chromatography on StrepTrap™ HP columns (GE Healthcare, Fairfield, CT, United States). The buffer was changed to PBS and endotoxin removed using the EndoTrap^®^ Endotoxin removal system (Hyglos GmbH, Bernried am Starnberger See, Germany). In order to avoid measuring an immune reaction against the Strep-tag, further restimulation experiments and ELISAs were conducted with C-terminal His-tagged SplB and GlpQ, that were purified the same way using HisTrap™ HP columns.

### Mice and Treatment Protocol

Female, *S. aureus*-naïve C57BL/6NRj (C57BL/6N) wild-type mice (Janvier Labs, Saint-Berthevin Cedex, France) were 8 weeks old during the start of the treatment protocol. Animals were kept under standard conditions, in open cages in an incubator, 12 h light/dark cycle, access to food and water *ad libitum*. All animal experiments were approved by the Landesamt für Landwirtschaft, Lebensmittelsicherheit und Fischerei Mecklenburg-Vorpommern (Az_7221.3-2-044_13).

Mice were primed with an intraperitoneal injection of 20 µg antigen in a physiological sodium chloride solution or in combination with either Imject™Alum (Thermo Fischer, Waltham, MA, United States) or Montanide ISA 71 VG (Seppic SA, Paris, France). Adjuvant-containing formulations were produced according to manufacturer`s instructions. When no antigen was added, adjuvants were mixed with physiological sodium chloride solution. Twenty-eight days after the first injection mice were boosted using the same formulations they received before. In both cases the intraperitoneal route of injection was chosen to elicit a systemic immune response and to promote self-drainage of the antigen and/or adjuvant to the lymphoid organs, e.g. the spleen (44). Seven days after boost mice were anesthetized with Ketamin/Xylazin (100 mg/10 mg per kg body weight, intraperitoneal), bled *via* retro-orbital puncture and euthanized by cervical dislocation. Afterward the spleen was removed under sterile conditions.

Blood was collected in sterile 1.5 mL reaction tubes and centrifuged for 10 min at 600 rcf. Serum was collected and stored at -80°C before further analysis. Splenocytes were isolated as described elsewhere (45).

### Flow Cytometry

The following antibodies were used for identification of immune cells directly after splenocyte isolation: Ly6G-BV421, CD11b-BV510, CD4-BV605, CD4-BV650, CD19-BV650, NK1.1-FITC, CD3-FITC, CD3-PerCP-Cy5.5, CD8-PE, CD11c-PE/Dazzle, I-A/I-E-PE/Cy7, Ly6C-AF647, GATA3-BV421, RORγT-PE (BD, Franklin Lakes, NJ, United States), FoxP3-APC (Miltenyi, Bergisch Gladbach, Germany), Tbet-PerCP-Cy5.5. All antibodies were purchased from Biolegend, San Diego, CA, United States, unless stated otherwise. After splenocyte isolation cells were washed with PBS and stained with Zombie NIR™ (Biolegend, San Diego, CA, United States) to mark dead cells. To avoid unspecific binding of antibodies Fc-receptors were blocked with an FcR Blocking Reagent (Miltenyi, Bergisch Gladbach, Germany). Afterward, cells were stained for 20 min at 4°C in the dark. Intranuclear stainings were performed using the True-Nuclear™ Transcription Factor Buffer Set (Biolegend, San Diego, CA, United States) according to manufacturer`s instructions. Cells were analyzed on a BD LSRII flow cytometer.

For intracellular cytokine staining 10^6^ splenocytes were seeded in 96 well plates and restimulated antigen-specifically (30 µg/mL) overnight at 37°C, 5% CO_2_. Culture was carried out in TexMACS™ medium (Miltenyi, Bergisch Gladbach, Germany), supplemented with 10% FCS, 1% Penicillin-Streptomycin-Glutamine (10,000 IU/mL, 10,000 µg/mL, 29.2 mg/mL; Thermo Fischer, Waltham, MA, United States) and 50 μM 2-mercaptoethanol. The next day, 0.1% BrefeldinA/Monensin (Biolegend, San Diego, CA, United States) was added and cells were cultured for an additional 4 hours. Afterward, cells were harvested and stained as described before. For fixation and permeabilization, Fixation Buffer and Intracellular Staining Permeabilization Wash Buffer (10X) (both Biolegend, San Diego, CA, United States) were used according to manufacturer`s instructions. The following antibodies were used for identification of immune cells: CD3-FITC, CD4-PerCP-Cy5.5, CD19-BV510, IL-10-PE, TNFα-PE/Cy7, IL-4-PE/Dazzle, IL-2-APC, IL-17-BV421, IFNγ-BV650 (all Biolegend, San Diego, CA, United States). Cells were analyzed on a BD LSRII flow cytometer.

### Cytokine and Chemokine Secretion Assay

2 × 10^5^ splenocytes were seeded and cultured as described in the previous section for 4 days. After culture, cell free supernatant was harvested and stored at -80°C before further analysis. The LEGENDplex™ Mouse Th Cytokine Panel (13-plex) and LEGENDplex™ Mouse Proinflammatory Chemokine Panel (13-plex) (both Biolegend, San Diego, CA, United States) were used according to manufacturer`s instructions for measuring cytokine and chemokine concentrations in the supernatant. Samples were analyzed on a BD LSRII flow cytometer.

### Antigen-Specific IgG, IgG1, IgG2c and IgE ELISA

96 well microtiter plates (Nunc MaxiSorp™, Affymetrix eBioscience, Santa Clara, CA, USA) were coated with 0.1 μg antigen per well (Sigma-Aldrich, St. Louis, MO, USA) in coating buffer (Candor Bioscience GmbH, Wangen, Germany) overnight at 4°C, washed with PBS/0.05% Tween20™ and blocked with Blocking Reagent (Sigma-Aldrich, St. Louis, MO, United States). For IgG determination, serum was diluted serially with a dilution factor of 4, starting at 1:200 and ending at 1:819,200; IgG1 and IgG2c determination started at 1:40 and ended at 1:163,840. IgG, IgG1 and IgG2c binding was detected using goat anti-mouse IgG, IgG1 or IgG2c coupled to HRP (all Southern Biotech, Birmingham, AL, USA) and BD OptEIA™ TMB Substrate Reagent Set (BD, Franklin Lakes, NJ, USA). Optical density at 450 nm was measured with the Tecan Sunrise photometer (Tecan Group Ltd., Maennedorf, Switzerland). The antigen-specific antibody titer (aU) was determined, as described elsewhere (36).

For measuring IgE levels, the process was adapted as follows: serum was diluted 1:6 in Blocking Reagent. Biotin-conjugated rat anti-murine IgE antibodies (BD, Franklin Lakes, NJ, USA) were used in combination with peroxidase-conjugated streptavidin (Dianova, Hamburg, Germany) to detect antibody binding. Single OD measurements were performed at 450 nm, and the blank value in the absence of serum was multiplied by 1.5 and subtracted.

### Statistical Analysis

Statistical analysis of results was carried out using GraphPad Prism 7.04 (Graphpad Software Inc., San Diego, CA, United States). The Kruskal–Wallis test was used followed by Dunn’s multiple comparison test to compare the treatment groups as follows: (i) Animals that received SplB or GlpQ without adjuvant; (ii) animals immunized with antigen only versus controls (NaCl) that had received no antigen; (iii) animals immunized with the same antigen with or without adjuvant; (iv) animals injected with physiological sodium chloride solution (NaCl) versus adjuvant-only animals. Results were considered statistically significant at *p < 0.05, **p < 0.01, ***p < 0.001, and ****p < 0.0001.

## Results

To determine the intrinsic immune polarization potential of the *S. aureus* antigens SplB and GlpQ, we immunized C57BL/6N mice by intraperitoneal injection of the native recombinant proteins without adjuvant. The animals were administered either SplB or GlpQ. Other experimental groups received the same antigens together with the adjuvants Montanide or Alum to find out how these modulate the antigens’ polarization potential. We used *S. aureus*-naïve animals throughout to ensure that the B cells and T cells had not encountered the antigens prior to the immunization (46, 47). Twenty-eight days after the priming immunization, we boosted the animals with the same formulation they had received for priming. Seven days after the boost immunization, we analyzed the immune response to the appropriate immunization antigens.

### Immunization With SplB Induced More Th1 and Th2 Cells Than GlpQ

**Figure 1:**
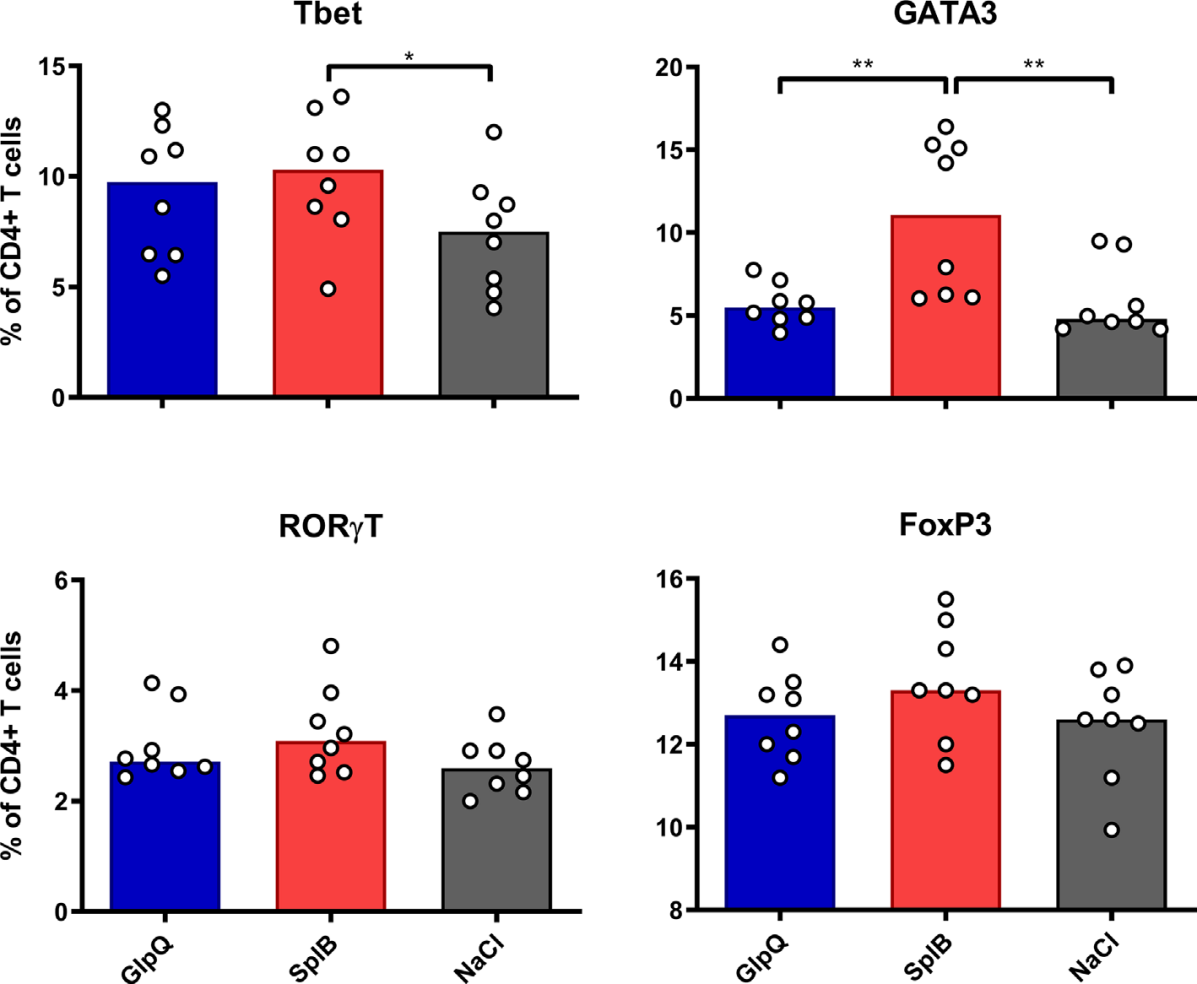
SplB induced Th2 cells. C57BL/6N mice were primed and boosted with non-adjuvanted antigen. Seven days after the boost, splenocytes were isolated and stained for Th1- (Tbet), Th2- (GATA3), Th17- (RORγT) and Treg- (FoxP3) specific transcription factors. Data are presented as median. n = 8 animals per group. *p < 0.05; **p < 0.01. Group comparisons that are defined in the “Statistical analysis” section but not shown here are not significant.

Immunization with SplB increased the proportions of Th1 (Tbet^+^) and Th2 (GATA3^+^) cells in the spleen significantly, but immunization with GlpQ did not (**Figure 1**). Neither Montanide nor Alum modulated the composition of the CD4^+^ T cell population in SplB-vaccinated mice (**Figure S1**), while animals that received Alum-adjuvanted GlpQ had a slight increase of regulatory T cells (Tregs, FoxP3^+^, **Figure S2**).

Next, we isolated splenocytes from vaccinated mice and controls, stimulated them overnight with the immunization antigen and determined the intracellular cytokines of the recall response *via* flow cytometry. The results matched the transcription factor patterns. Following immunization with SplB, the percentages of IL-4- and IL-10-expressing CD4^+^ T cells, typical of a Th2 polarization, more than doubled. The SplB-treated group also showed higher proportions of TNFα- and IL-2-expressing CD4^+^ T cells, which are characteristic of a Th1 profile. This effect was less pronounced than the Th2 response (**Figure S3A**). In contrast, immunization with GlpQ did not increase the T cells’ cytokine response to antigen restimulation *ex vivo* significantly (**Figure S3B**).

Montanide and Alum increased the proportion of CD4^+^ T cells with intracellular cytokine expression (except IL-2), regardless of whether the animals had received a protein antigen or not (**Figure S3**). The effects of SplB or GlpQ and Montanide appeared to be additive, whereas Alum augmented the response in mice that had received GlpQ but not in those immunized with SplB.

The transcription factor- and cytokine profiles show that immunization with SplB alone – but not with GlpQ – upregulated both Th1 and Th2 cells in the spleen. Adjuvant treatment generally increased the T cells’ reaction potential, and it often boosted their antigen-specific cytokine responses to restimulation in cell culture.

### Immunization With SplB Facilitated the Release of Type 2 Cytokines

**Figure 2:**
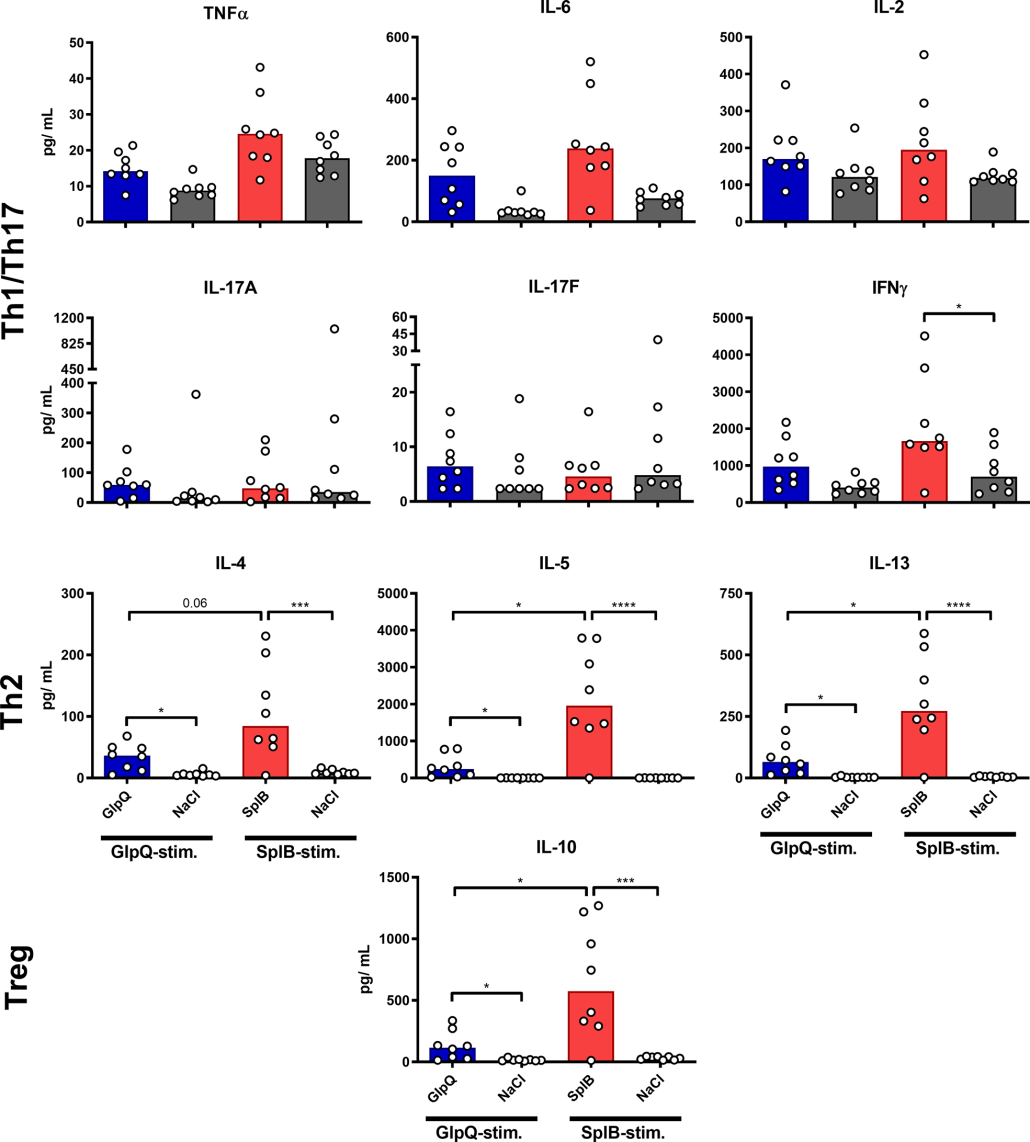
SplB but not GlpQ promoted Th2 cytokine production. Isolated splenocytes were restimulated with the indicated vaccine antigen for 4 days, afterward the supernatant was harvested and the concentration of produced cytokines was measured *via* the LEGENDplex™ Mouse Th Cytokine Panel (13-plex). Data are presented as median. n = 8 animals per group. *p < 0.05; ***p < 0.001; ****p < 0.0001. Group comparisons that are defined in the “Statistical analysis” section but not shown here are not significant.

To determine the reaction potential of the splenocytes after the prime-boost immunization, we restimulated them with antigen in cell culture for 4 days and determined the cytokine concentrations in the supernatants. Splenocytes from immunized animals (without adjuvant) produced significantly more type 2 cytokines – IL-4, IL-5, IL-10 and IL-13 – than those from non-immunized controls. The effect was significantly stronger in animals immunized with SplB than in the GlpQ-group (**Figure 2**). With respect to Th1/Th17 cytokines, only IFNγ was significantly higher in SplB-treated mice compared to control animals. IL-17A, IL-17F and IFNγ increased slightly, but not significantly, following immunization with GlpQ.

**Figure 3:**
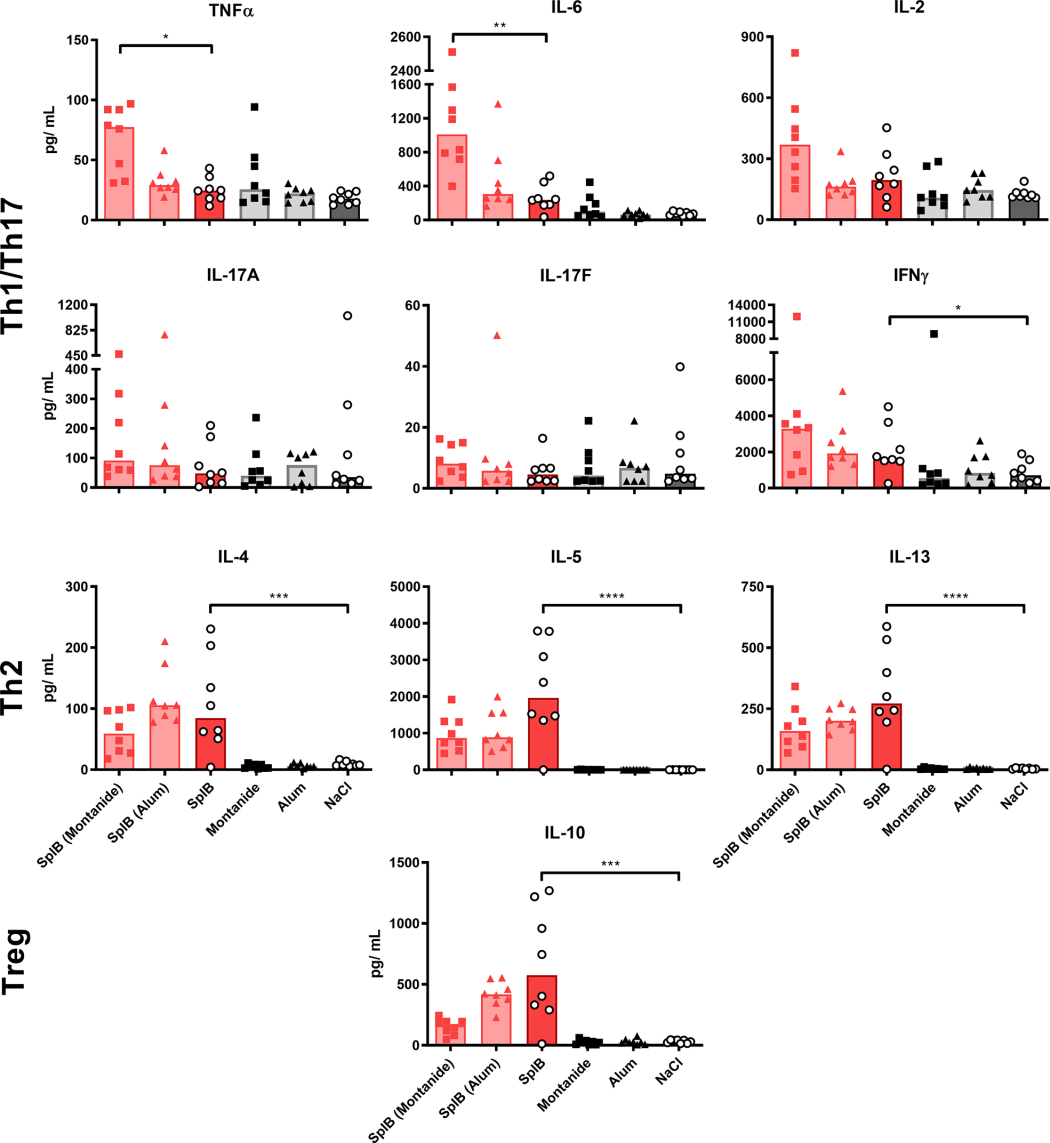
Adjuvants had little effect on cytokine production of SplB-treated animals. Isolated splenocytes were restimulated with SplB for 4 days, afterward the supernatant was harvested and the concentration of produced cytokines was measured *via* the LEGENDplex™ Mouse Th Cytokine Panel (13-plex). Data are presented as median. n = 8 animals per group. *p < 0.05; **p < 0.01; ***p < 0.001; ****p < 0.0001. Group comparisons that are defined in the “Statistical analysis” section but not shown here are not significant.

**Figure 4:**
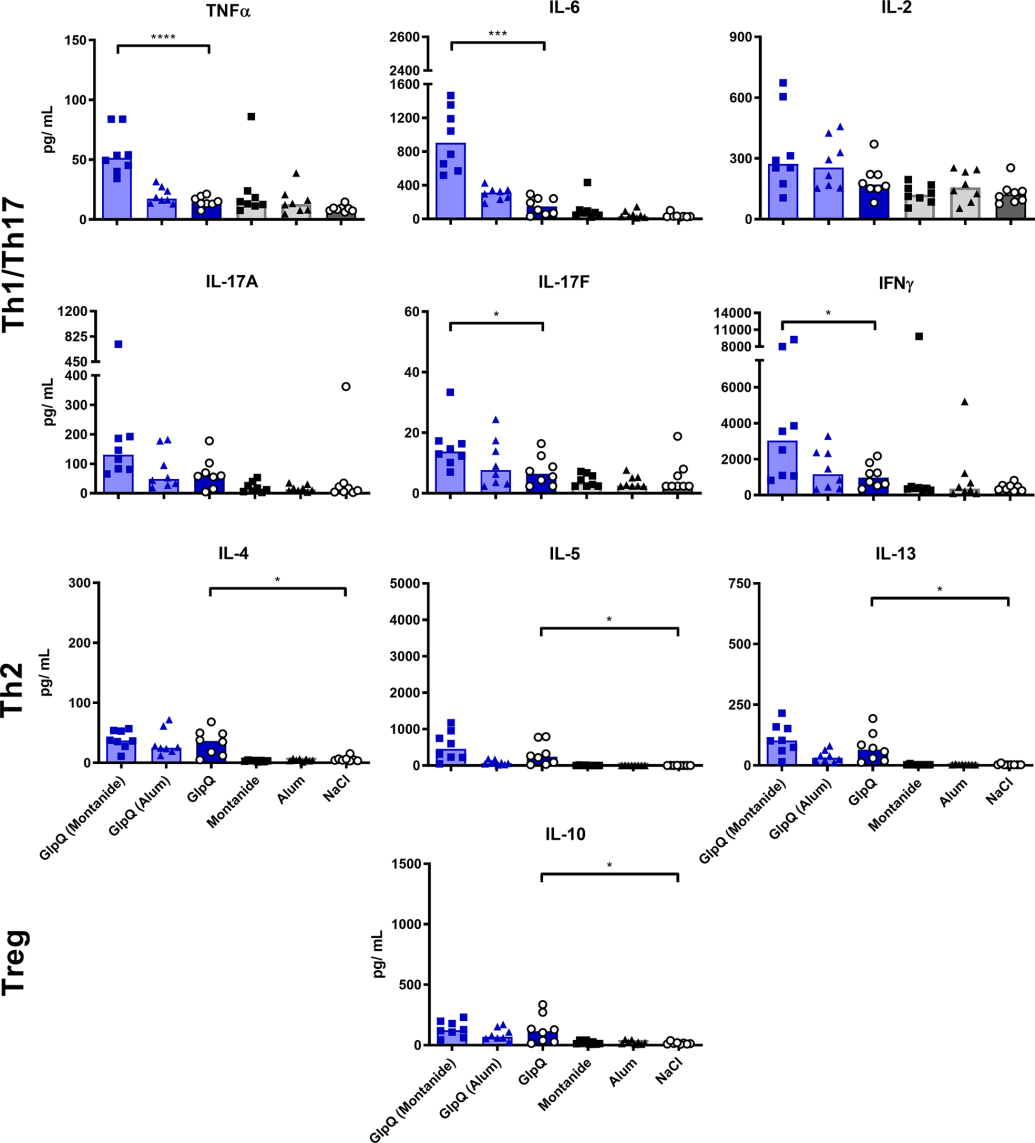
Montanide increased Th1/Th17 cytokine production. Isolated splenocytes were restimulated with GlpQ for 4 days, afterward the supernatant was harvested and the concentration of produced cytokines was measured *via* the LEGENDplex™ Mouse Th Cytokine Panel (13-plex). Data are presented as median. n = 8 animals per group. *p < 0.05; ***p < 0.001; ****p < 0.0001. Group comparisons that are defined in the “Statistical analysis” section but not shown here are not significant.

Montanide selectively boosted the production of Th1/Th17 cytokines, irrespective of the used antigen. The effect was significant for TNFα, IL-6 and IL-17F. In contrast, Th2 cytokines were not affected by Montanide; they even tended to decrease in animals immunized with SplB. Alum had no significant effect on the cytokine release. Application of adjuvant alone did not increase the splenocytes’ cytokine release upon antigen re-stimulation *in vitro* (**Figures 3** and **4**).

**Figure 5:**
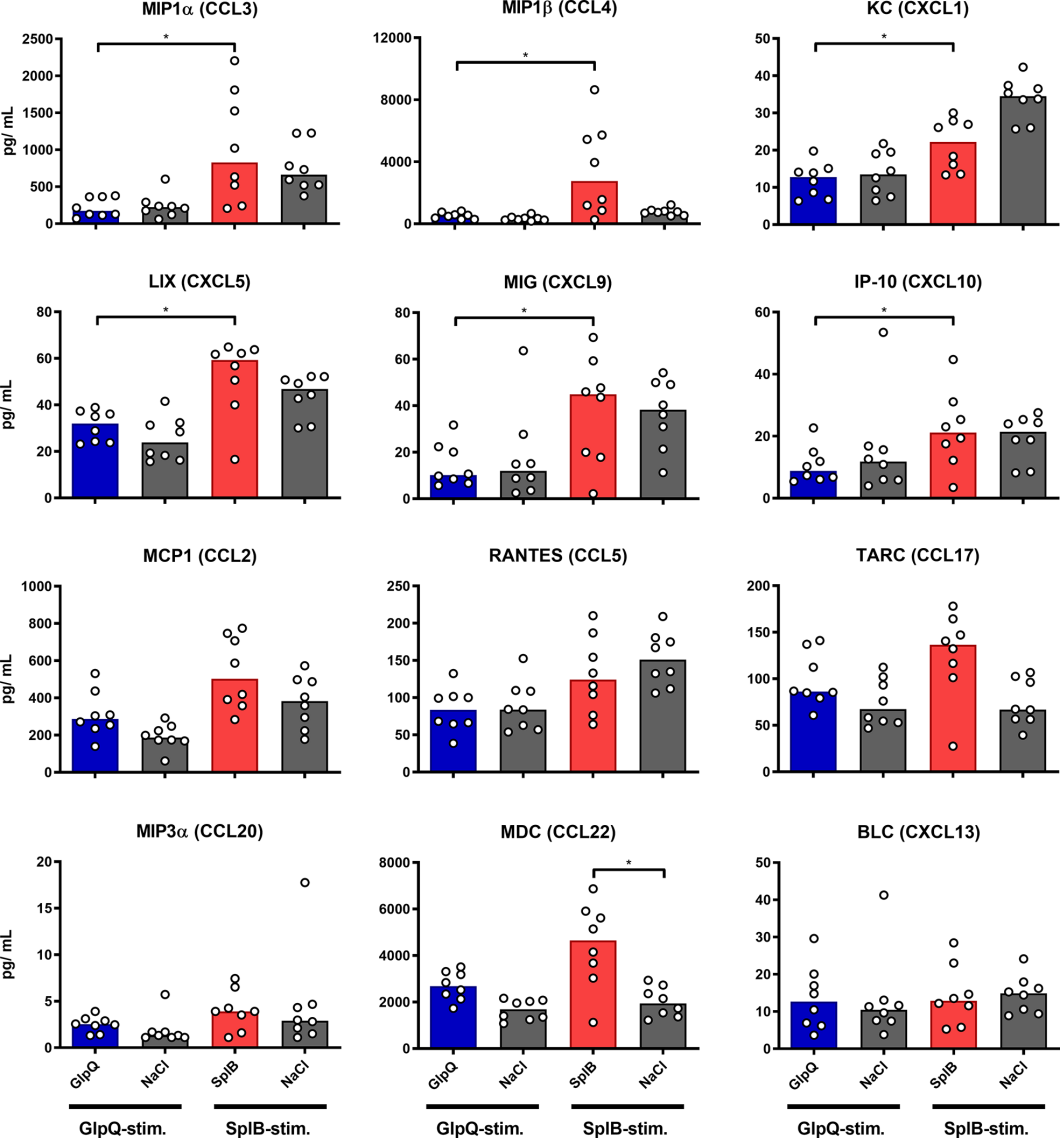
SplB provoked a stronger production of proinflammatory chemokines than GlpQ. Isolated splenocytes were restimulated with the indicated vaccine antigen for 4 days, afterward the supernatant was harvested and the concentration of produced chemokines was measured *via* the LEGENDplex™ Mouse Proinflammatory Chemokine Panel (13-plex). Data are presented as median. n = 8 animals per group. *p < 0.05. Group comparisons that are defined in the “Statistical analysis” section but not shown here are not significant.

Looking at chemokines, SplB-treated animals produced significantly more MIP1α, MIP1β, KC, LIX, MIG and IP-10 than the mice in the GlpQ-group, which fits the type 2 profile of the immune response to SplB. Even splenocytes from NaCl-control animals tended to react to SplB exposure in cell culture with chemokine release. MIP1α, MIP1β and KC are strongly associated with a type 2 profile whereas MIG and IP-10 are linked to a Th1 phenotype (48–50) (**Figure 5**).

**Figure 6:**
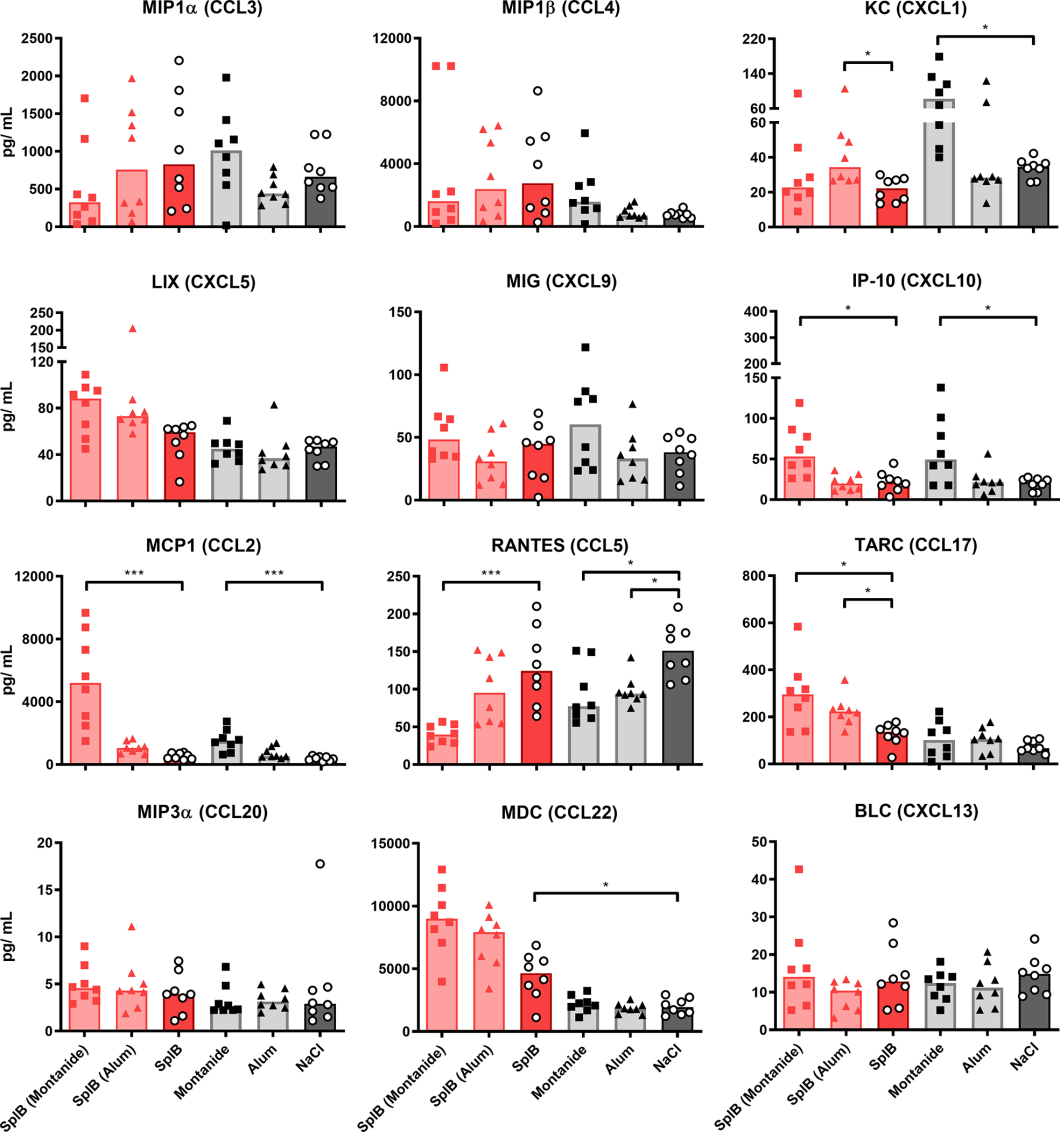
Montanide-adjuvanted SplB-animals produced more IP-10, MCP1 and TARC. Isolated splenocytes were restimulated with SplB for 4 days, afterward the supernatant was harvested and the concentration of produced chemokines was measured *via* the LEGENDplex™ Mouse Proinflammatory Chemokine Panel (13-plex). Data are presented as median. n = 8 animals per group. *p < 0.05; ***p < 0.001. Group comparisons that are defined in the “Statistical analysis” section but not shown here are not significant.

**Figure 7:**
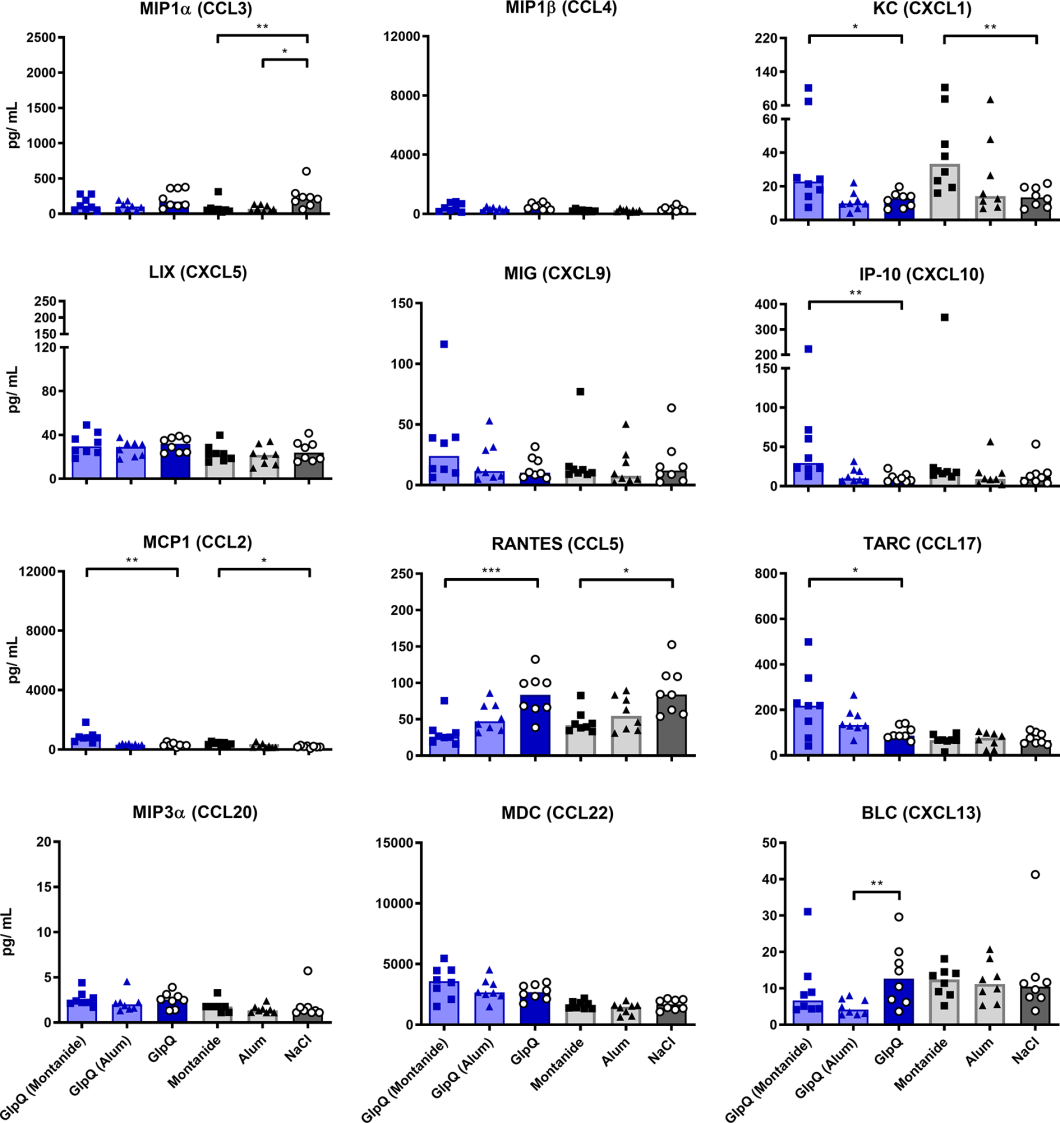
GlpQ restimulation provoked little chemokine production. Isolated splenocytes were restimulated with GlpQ for 4 days, afterward the supernatant was harvested and the concentration of produced chemokines was measured *via* the LEGENDplex™ Mouse Proinflammatory Chemokine Panel (13-plex). Data are presented as median. n = 8 animals per group. *p < 0.05; **p < 0.01; ***p < 0.001. Group comparisons that are defined in the “Statistical analysis” section but not shown here are not significant.

The addition of Montanide to either antigen increased the induction of KC, IP-10, MCP1 and TARC *ex vivo*, while RANTES was strongly reduced. The former chemokines are important for the trafficking of neutrophils, NK cells, monocytes and T cells, respectively, while RANTES plays an active role in recruiting T cells, macrophages, eosinophils and basophils (51, 52). Alum increased the production of KC and TARC when given in combination with SplB and decreased the production of BLC, when given in combination with GlpQ (**Figures 6** and **7**).

Thus, administration SplB alone promoted the generation of cells that respond to restimulation with the production of type 2-associated cytokines and chemokines. This was much less pronounced in animals that had received GlpQ.

### The Antibody Response to SplB Reflected the Cellular Type 2 Bias

To see how the cellular immune response translates into humoral immunity, we measured antigen-specific serum antibodies 7 days after the boost immunization.

**Figure 8:**
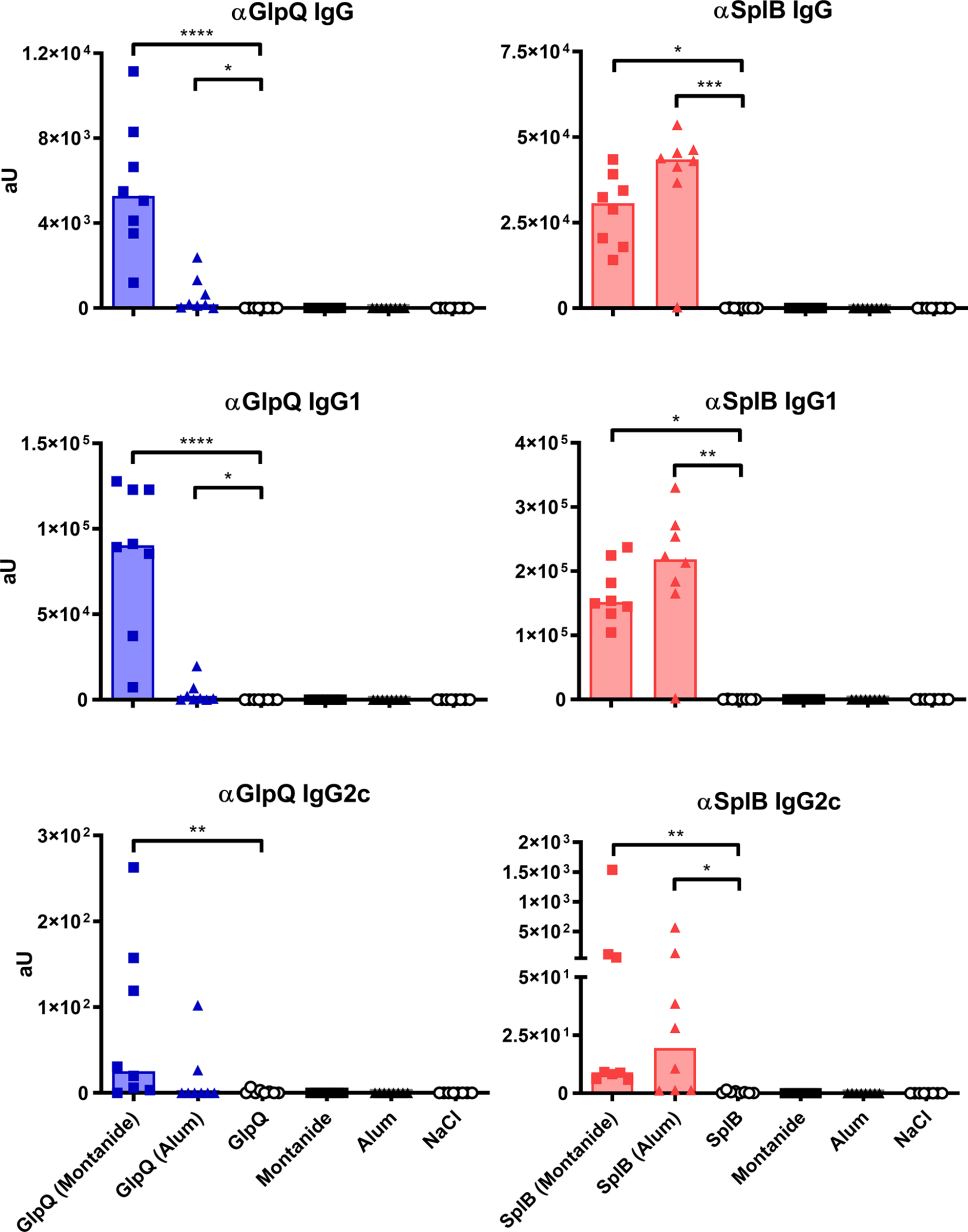
Alum and Montanide affected antigen-specific IgG production differently. Antigen-specific IgG, IgG1 and IgG2c were measured in the serum 7 days after boost *via* ELISA. Data are presented as median. n = 8 animals per group. *p < 0.05; **p < 0.01; ***p < 0.001; ****p < 0.0001. Group comparisons that are defined in the “Statistical analysis” section but not shown here are not significant.

Immunization with SplB or GlpQ alone was not sufficient for the induction of antigen-specific IgG, confirming that the animals had not been exposed to *S. aureus* prior to the immunization. In animals treated with SplB, Alum increased the antigen-specific IgG levels maximally, but it failed to induce a specific IgG response to GlpQ. Addition of Montanide triggered a strong specific IgG response to both antigens. Since IgG1 and IgG2c are associated with Th2 or Th1 responses, respectively (53, 54), we expected an antigen effect on their production; however, the antigen-specific IgG1- and IgG2c- concentrations showed the same patterns as the total specific serum IgG (**Figure 8**).

**Figure 9:**
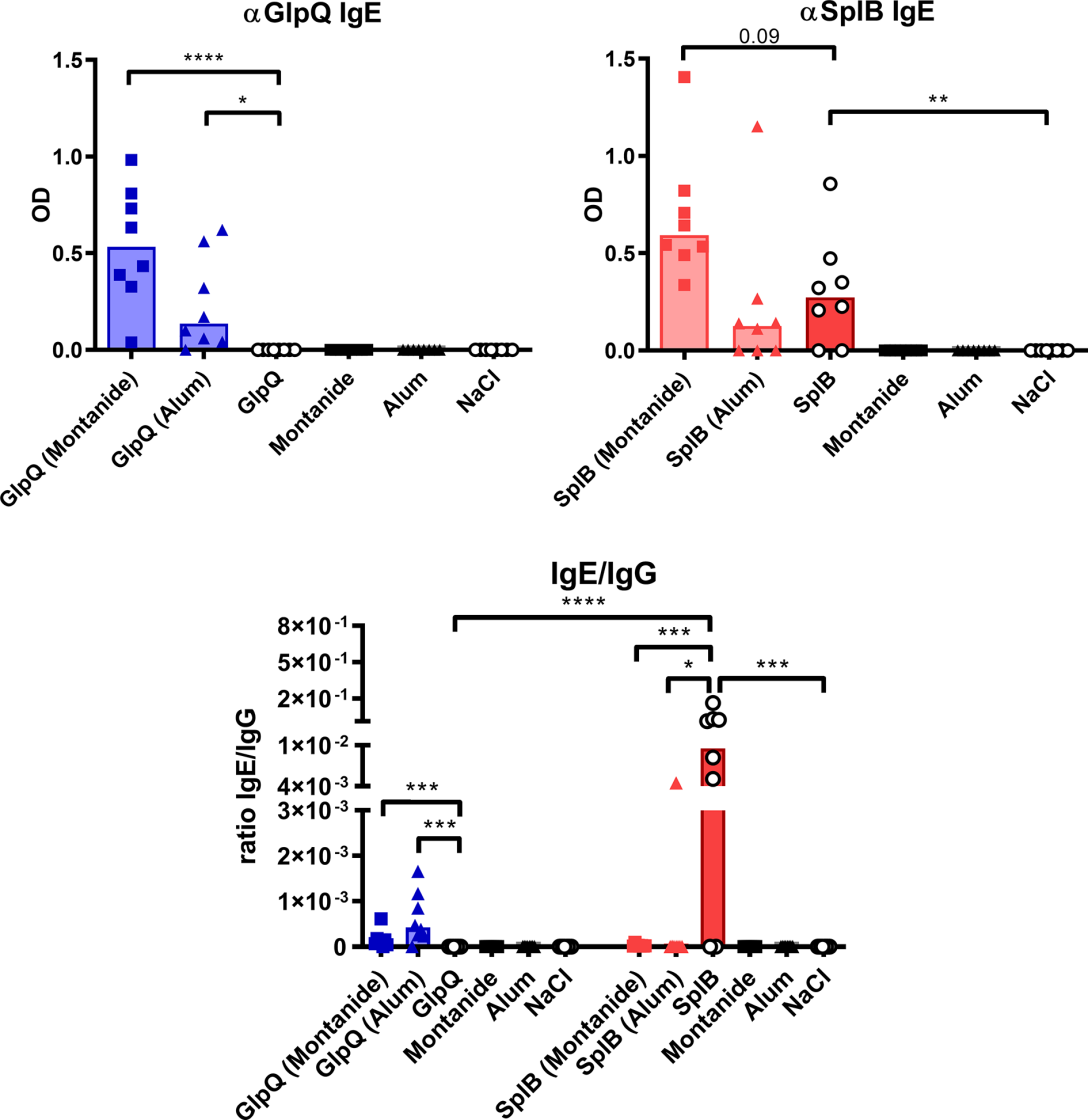
Non-adjuvanted SplB induced IgE production and skewed the antibody response toward a type 2 profile. Antigen-specific IgE was measured in the serum 7 days after boost *via* ELISA and compared to the measured IgG response. Data are presented as median. n = 8 animals per group. OD: optical density. *p < 0.05; **p < 0.01; ***p < 0.001; ****p < 0.0001. Group comparisons that are defined in the “Statistical analysis” section but not shown here are not significant.

Remarkably, immunization with SplB alone induced specific IgE, which GlpQ did not (**Figure 9**). Montanide always boosted IgE production, whereas Alum increased IgE production marginally when given with GlpQ but did not further enhance the SplB-specific IgE. The values of the IgE/IgG ratios underline how strongly SplB skewed the antibody response toward a type 2 profile (**Figure 9**). In conclusion, the humoral immune response also reflects the type 2 bias in the immune response to SplB of *S. aureus*.

### The Numbers of Unconventional Antigen-Presenting Cells in the Spleen Correlated With the Antibody Production

To find out which cell type might be responsible for the type 2 polarization of the immune reaction to SplB, we turned to antigen-presenting cells in the spleen. We analyzed dendritic cells (DCs) and B cells as well as unconventional antigen-presenting cells: neutrophils, eosinophils and inflammatory monocytes.

**Figure 10:**
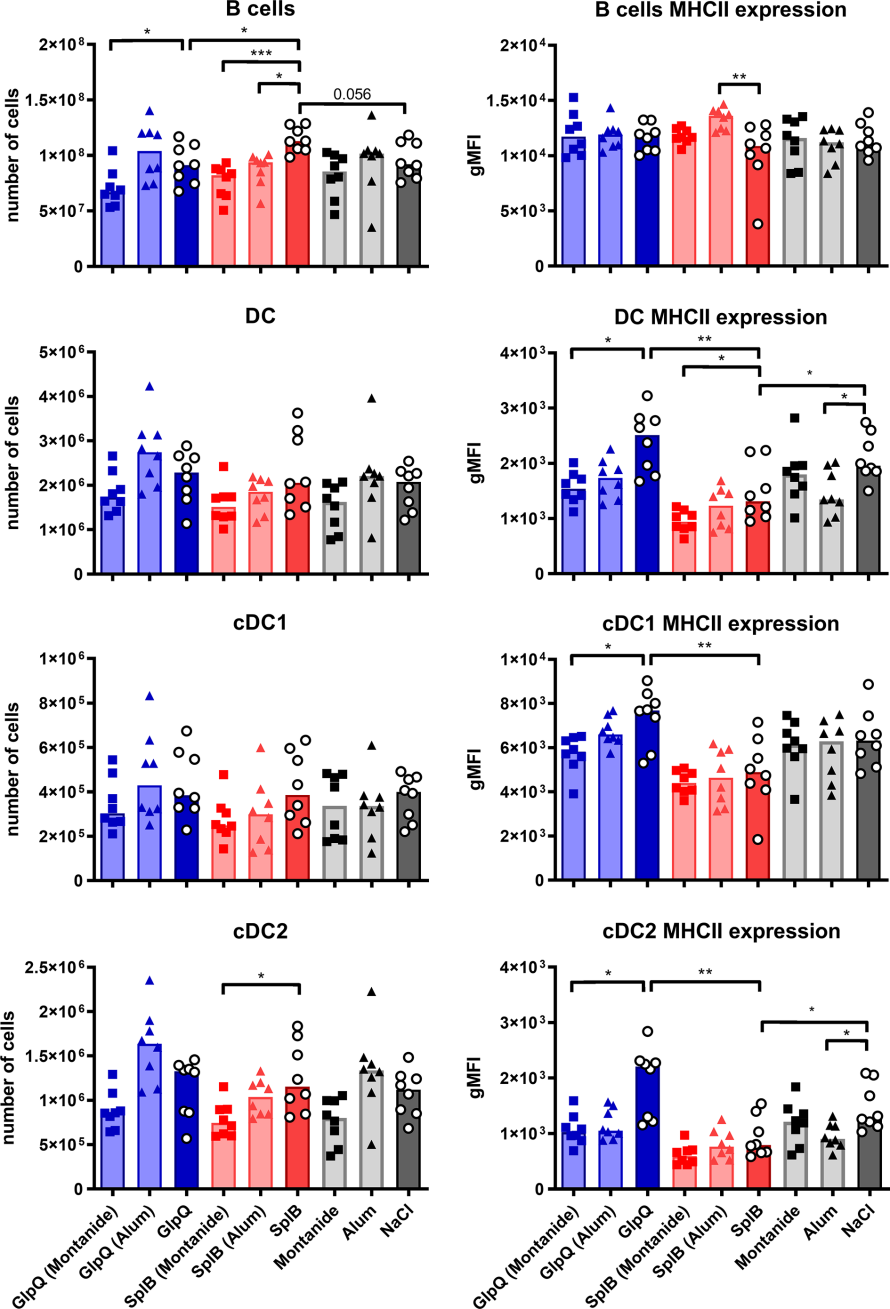
Moderate effects of antigens or adjuvants on conventional antigen-presenting cells. C57BL/6N mice were primed and boosted with either non-adjuvanted or adjuvanted antigen. Seven days after the boost, splenocytes were isolated and B cells, DCs, cDC1 and cDC2 enumerated and characterized with respect to MHC-II expression. Data are presented as median. gMFI: geometric mean fluorescence intensity. n = 8 animals per group. *p < 0.05; **p < 0.01; ***p < 0.001. Group comparisons that are defined in the “Statistical analysis” section but not shown here are not significant.

Animals immunized with SplB alone had slightly more splenic B cells than GlpQ-immunized animals or controls. The numbers of DCs, however, did not differ significantly between immunized and control animals. The same was true for their subtypes, cDC1 and cDC2, that are associated with Th1 or Th2 responses, respectively. However, MHC-II expression on all DC subpopulations was higher following immunization with GlpQ than with SplB (**Figure 10**).

Addition of Montanide reduced the expression of MHC-II on DCs, especially in the GlpQ group, and Alum tended to do the same. Otherwise, the adjuvants had only minor effects on conventional antigen-presenting cells (**Figure 10**).

**Figure 11:**
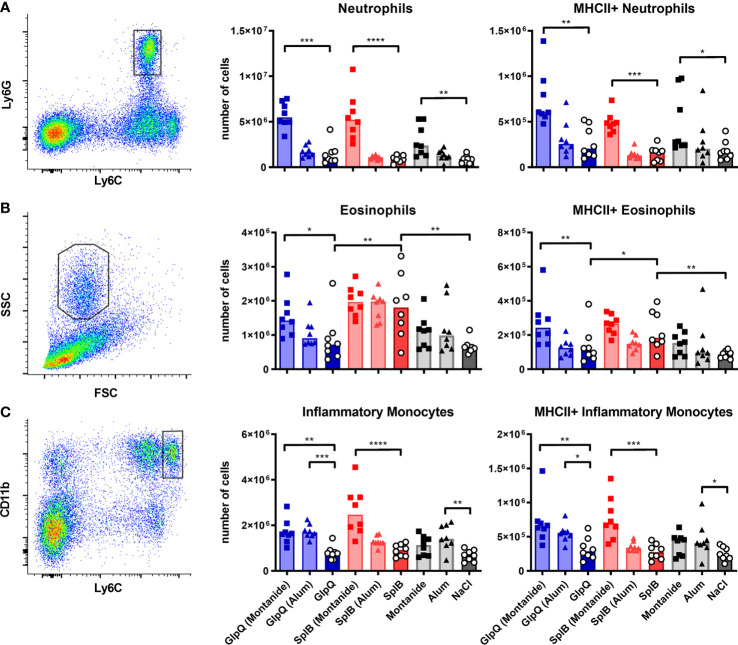
Montanide increased the number of unconventional antigen-presenting cells; SplB elicited an eosinophil response. C57BL/6N mice were primed and boosted with either non-adjuvanted or adjuvanted antigen. Seven days after the boost, splenocytes were isolated and different unconventional antigen-presenting cells were enumerated and characterized with respect to MHC-II expression. **(A)** Neutrophils were defined as Ly6C^+^/Ly6G^+^ cells. **(B)** After the exclusion of neutrophils and monocytes, eosinophils were defined as FSC^int^/SSC^high^ cells. **(C)** After the exclusion of lymphocytes, inflammatory monocytes were defined as Ly6C^high^/CD11b^high^ cells. Data are presented as median. n = 8 animals per group. *p < 0.05; **p < 0.01; ***p < 0.001; ****p < 0.0001. Group comparisons that are defined in the “Statistical analysis” section but not shown here are not significant.

Since neither the absolute numbers nor the MHC-II expression nor DC polarization correlated with the antibody production, we next turned to unconventional antigen-presenting cells that are capable of antigen presentation besides other main functions. Neutrophils and eosinophils can act as antigen-presenting cells; the latter are usually associated with a type 2 immune response (55, 56). Inflammatory monocytes can differentiate into DCs, present antigen and efficiently activate T cells, thereby promoting antibody generation (40). Exposure to SplB alone strongly increased numbers of eosinophils in the spleen seven days after the boost immunization. The cells were activated with elevated MHC-II expression. The eosinophils did not respond if GlpQ was the immunizing antigen (**Figure 11B**). These findings extend the results of the cytokine- and antibody measurements and underline the distinctive polarization potentials of SplB and GlpQ of *S. aureus*.

Montanide had a prominent effect on splenic neutrophils and increased their numbers and MHC-II expression strongly. Eosinophils and inflammatory monocytes were similarly affected, albeit to a lesser extent (**Figure 11**). This correlated with the IgG response. Alum had little effect; it only increased numbers and activation of inflammatory monocytes when given alone or together with GlpQ (**Figure 11C**).

## Discussion

We have shown that the immune system of *S. aureus*-naïve C57BL/6N mice responded differently to the *S. aureus* antigens GlpQ and SplB. SplB polarized the immune response toward a type 2 response, whereas there was no clear polarization in GlpQ-treated animals. The adjuvants also had different effects on the immune system. Montanide induced inflammation of a Th1/Th17 profile and strongly increased the antigen-specific IgG production. Alum, on the other hand, increased the IgG production selectively in the SplB-treated mice.

We suspected that the Spls of *S. aureus* have type 2 immune polarizing, i.e., allergenic potential, because humans that are naturally exposed to the bacteria develop an Spl-specific immune response that is characterized by IgE and IgG4 as well as by Th2 cytokines. Moreover, SplD induces asthma in mice if applied intratracheally without adjuvant (36, 57, 58). The results of our murine intraperitoneal immunization experiments support the idea that SplB has intrinsic type 2 polarizing potential. In our murine model the immune response to the intraperitoneal application of SplB (alone) was not biased by adjuvants and, unlike the lung, the peritoneum is no Th2-promoting micro-environment (23). By applying recombinant purified SplB, we also avoided the presence of other *S. aureus* antigens and/or PAMPs that likely influence the immune response profile during colonization, infection or immunization. In this setting SplB induced Th2 cells and specific IgE *in vivo* as well as type 2 cytokines and chemokines in a recall response *ex vivo*, clearly demonstrating its type 2 polarizing potential. In addition, the immune response to SplB had some characteristics of a type 1 response, albeit less pronounced. There was some induction of Th1 cells as well as production of TNFα, IFNγ and the chemokines MIG and IP-10 that are typical of a type 1 profile. However, MIG and IP-10 can also be produced by eosinophils (46–48), and these cells are hallmarks of a type 2 inflammation. Their numbers were increased in the spleens of SplB-immunized animals. Mixed responses to a single antigen are common in humans (35, 37). With respect to *S. aureus*, type 2 dominance in combination with TNFα and IFNγ release has also been observed in the reaction of CD8^+^ T cells to protein A (SpA) and penicillin binding protein 2a (PBP2a) (59). It is further possible that SplB contains both Th1 and Th2 polarizing epitopes as it was shown for antigens of *Helicobacter pylori* (60), *Cryptococcus neoformans* (29) and humans (28, 61). Bystander activation of unrelated T cells as a consequence of vaccine-induced inflammation could be yet another reason for the Th1 aspects of the immune response to SplB (62).

In naturally exposed humans GlpQ was reported as a Th1/Th17-associated antigen (35). In our mouse model, however, GlpQ was only weakly immunogenic.

Immunomodulatory properties of an antigen may directly influence the profile of a vaccine response as demonstrated by our model. The simple approach, intraperitoneal application of a native antigen, may help to determine the intrinsic polarizing potential of vaccine candidates. For this purpose, the immune system of the experimental animals must be naïve to the test antigen. Many laboratory mice are colonized with *S. aureus* (46, 47). These would be unsuitable for this approach because the colonizing bacteria may have already polarized the immune response as it has been observed in humans, who are naturally exposed to *S. aureus* (35, 36). Similar to the polarizing potential of a vaccine antigen, pre-existing immunity could affect the vaccine response and polarize it (63). This may be relevant for the vaccine effect. Mouse models have shown that Th1 responses are protective in systemic *S. aureus* infections, while Th2 responses are of little help (18, 19). Therefore, the polarizing potential and the profile of the pre-existing specific immune response could be relevant for the selection of vaccine candidates.

Adjuvants are used to (i) enhance the vaccine response to weakly immunogenic antigens and (ii) direct it toward the desired immune profile. Therefore, we analyzed the immunomodulatory properties of Alum and Montanide in our model.

Alum is known as a Th2-promoting adjuvant. Aluminum-based adjuvants have usually little effect on the cellular immune response, but enhance antibody production (64). This is corroborated by our results, where Alum strongly increased the production of IgG specific for the type 2 polarizing antigen SplB but only very small amounts of GlpQ-specific IgG. Alum had low impact on the T cell polarization and cytokine or chemokine production. It is noteworthy, that the strongest antibody production occurred when the Th2-polarizing antigen SplB was combined with the Th2-polarizing adjuvant Alum. The adjuvant function of Alum is also influenced by the adsorption rate, adsorption strength and other properties of the antigen (40, 65). These were not analyzed in this study. Alum induced GlpQ-specific IgE production but did not increase the – much higher – baseline concentrations of SplB-specific IgE. We do not know the reasons for this difference. In terms of antigen-presenting cells our data are consistent with a report by the group of He who described that Alum increases the number of inflammatory monocytes that can differentiate into DCs and promote antibody production (40).

Montanide was designed to raise the Th1 response and improve IgG production, especially in antigens of low immunogenicity (43). Indeed, Montanide enhanced the production of type 1/3 cytokines, independent of the co-administered antigen, thereby generating an inflammatory environment. In animals immunized with GlpQ Montanide induced Th1/Th17 cytokines. In the SplB-immunized group, Montanide tended to reduce the production of Th2 cytokines, but was not able to override the Th2 bias or significantly decrease Th2 cell- or eosinophil numbers.

Montanide but not Alum strongly boosted the IgG response to immunization with GlpQ. In the SplB-group, IgG production was also increased by Montanide, but Alum was more effective. Since Montanide had a negative effect on the numbers of B cells and DCs, we suspect that unconventional antigen-presenting cells may have contributed to the increased antibody production. Their numbers in the spleen increased in the presence of Montanide, and they were activated. Unexpectedly, Montanide also increased the IgE production specific for both GlpQ and SplB. Apparently Montanide does not polarize the humoral response but rather increases antibody production in general. Increased IgE production has also been observed with Montanide ISA 51 VG, which is based on mineral-oil and designed to make a water-in-oil emulsion like Montanide ISA 71 VG that was used in this study (66).

In summary, our study shows that a simple intraperitoneal immunization model in antigen-naïve C57BL/6N mice can help to determine the intrinsic immunogenicity and immune polarization potential of antigens. This may be useful for characterizing vaccine candidates. The *S. aureus* protein SplB had a type 2 polarizing potential, consistent with observations in humans. GlpQ was poorly immunogenic. Alum selectively increased IgG production in response to the type 2 polarizing antigen SplB, but had little effect on immune cells. Montanide significantly enhanced the antibody response and increased inflammation, but could neither polarize the reaction to GlpQ nor reprofile the Th2 response induced by SplB. This indicates that intrinsic immune modulating properties of bacterial proteins can manifest themselves even in the presence of adjuvants (30).

## Data Availability Statement

The original contributions presented in the study are included in the article/**Supplementary Material**. Further inquiries can be directed to the corresponding author.

## Ethics Statement

The animal study was reviewed and approved by Landesamt für Landwirtschaft, Lebensmittelsicherheit und Fischerei Mecklenburg-Vorpommern (Az_7221.3-2-044_13).

## Author Contributions

DM designed, planned and performed experiments, analyzed the data, interpreted the results, and wrote the manuscript. PT, IJ, GD, and CB performed experiments. BB interpreted the results and edited the manuscript. All authors contributed to the article and approved the submitted version.

## Funding

This work was supported by the Bundesministerium für Bildung und Forschung (BB, DM, InfectControl2020, project InVAC, FKZ 03ZZ0806B, https://www.bmbf.de/), the Deutsche Forschungsgemeinschaft (PT, BB FKZ CRC-TRR34, RTG 1870, http://www.dfg.de/) and the State of Mecklenburg Western Pomerania (BB; ESF project KoInfekt, FKZ ESF/14-BM-A55-0004/16).

## Conflict of Interest

The authors declare that the research was conducted in the absence of any commercial or financial relationships that could be construed as a potential conflict of interest.
