# Supplementary material for: Immune Polarization Potential of the S. aureus Virulence Factors SplB and GlpQ and Modulation by Adjuvants
Source: Front Immunol. 2021 Apr 15;12:642802. doi: 10.3389/fimmu.2021.642802 (PMC8081891; doi:10.3389/fimmu.2021.642802)
Supplement: Supplementary file 1 [file Image_1.pdf]

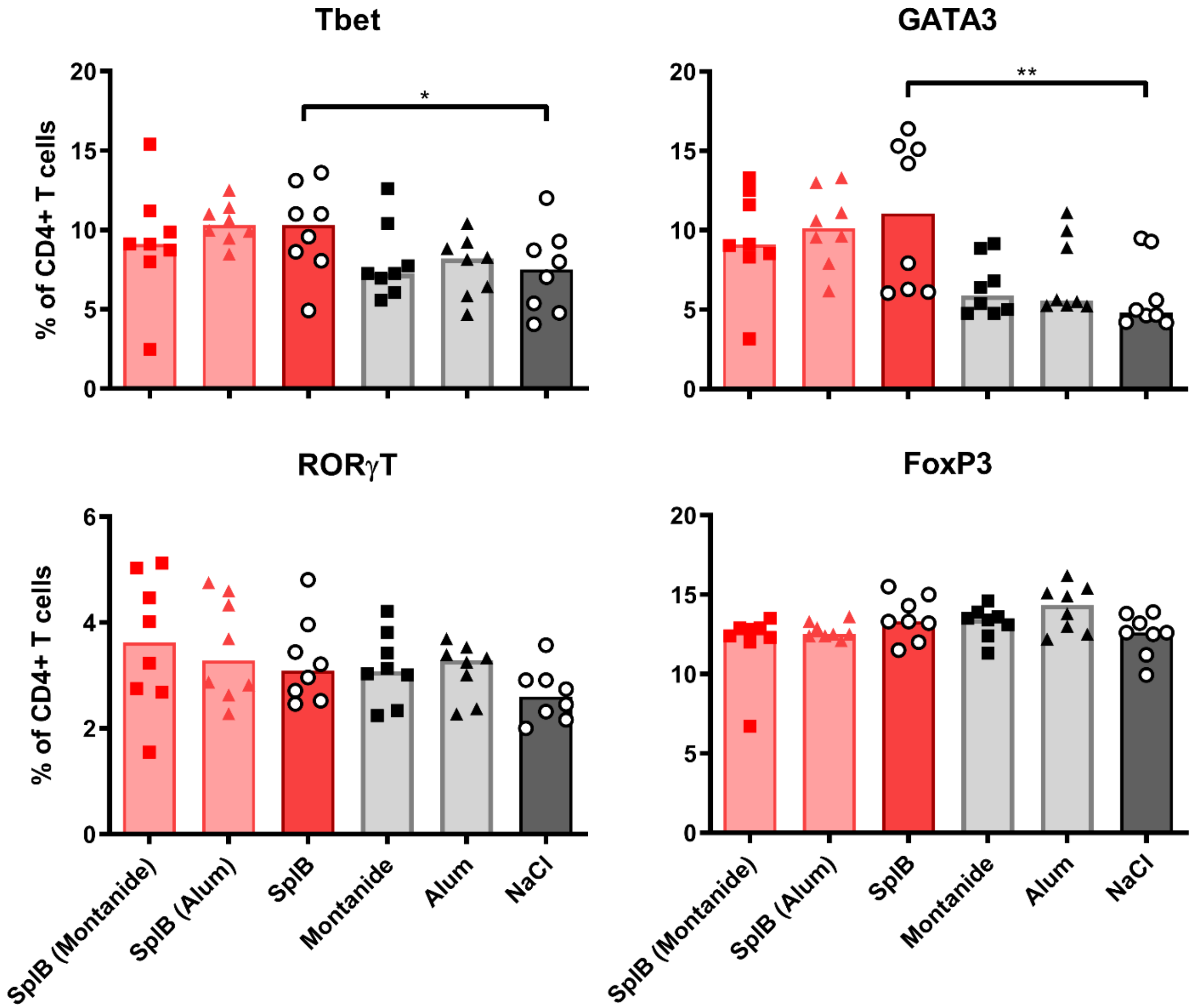

Supplementary Figure 1. SplB induced Th2 cells. C57BL/6N mice were primed and boosted with either non-adjuvanted or adjuvanted SplB. Seven days after the boost, splenocytes were isolated and stained for Th1- (Tbet), Th2- (GATA3), Th17- (RORγT) and Treg- (FoxP3) specific transcription factors. Data are presented as median. n = 8 animals per group. *$p < 0.05$; **$p < 0.01$.
