# Supplementary material for: Immune Polarization Potential of the S. aureus Virulence Factors SplB and GlpQ and Modulation by Adjuvants
Source: Front Immunol. 2021 Apr 15;12:642802. doi: 10.3389/fimmu.2021.642802 (PMC8081891; doi:10.3389/fimmu.2021.642802)
Supplement: Supplementary file 2 [file Image_2.pdf]

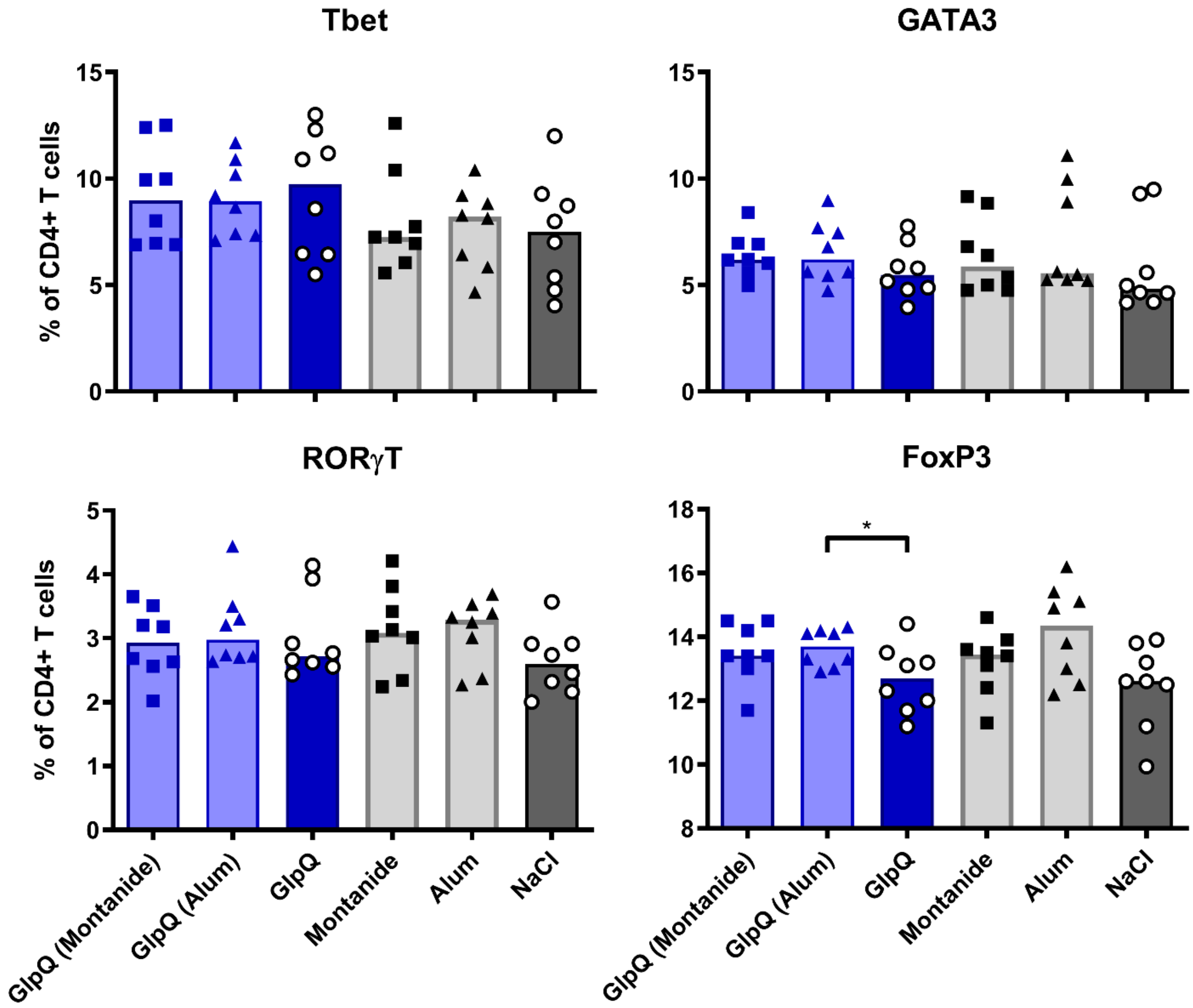

Supplementary Figure 2. Transcription factors in CD4⁺ T cells of GlpQ-immunized mice. C57BL/6N mice were primed and boosted with either non-adjuvanted or adjuvanted GlpQ. Seven days after the boost, splenocytes were isolated and stained for Th1- (Tbet), Th2- (GATA3), Th17- (RORγT) and Treg- (FoxP3) specific transcription factors. Data are presented as median. n = 8 animals per group. *p < 0.05.
