# Supplementary material for: Immune Polarization Potential of the S. aureus Virulence Factors SplB and GlpQ and Modulation by Adjuvants
Source: Front Immunol. 2021 Apr 15;12:642802. doi: 10.3389/fimmu.2021.642802 (PMC8081891; doi:10.3389/fimmu.2021.642802)
Supplement: Supplementary file 3 [file Image_3.pdf]

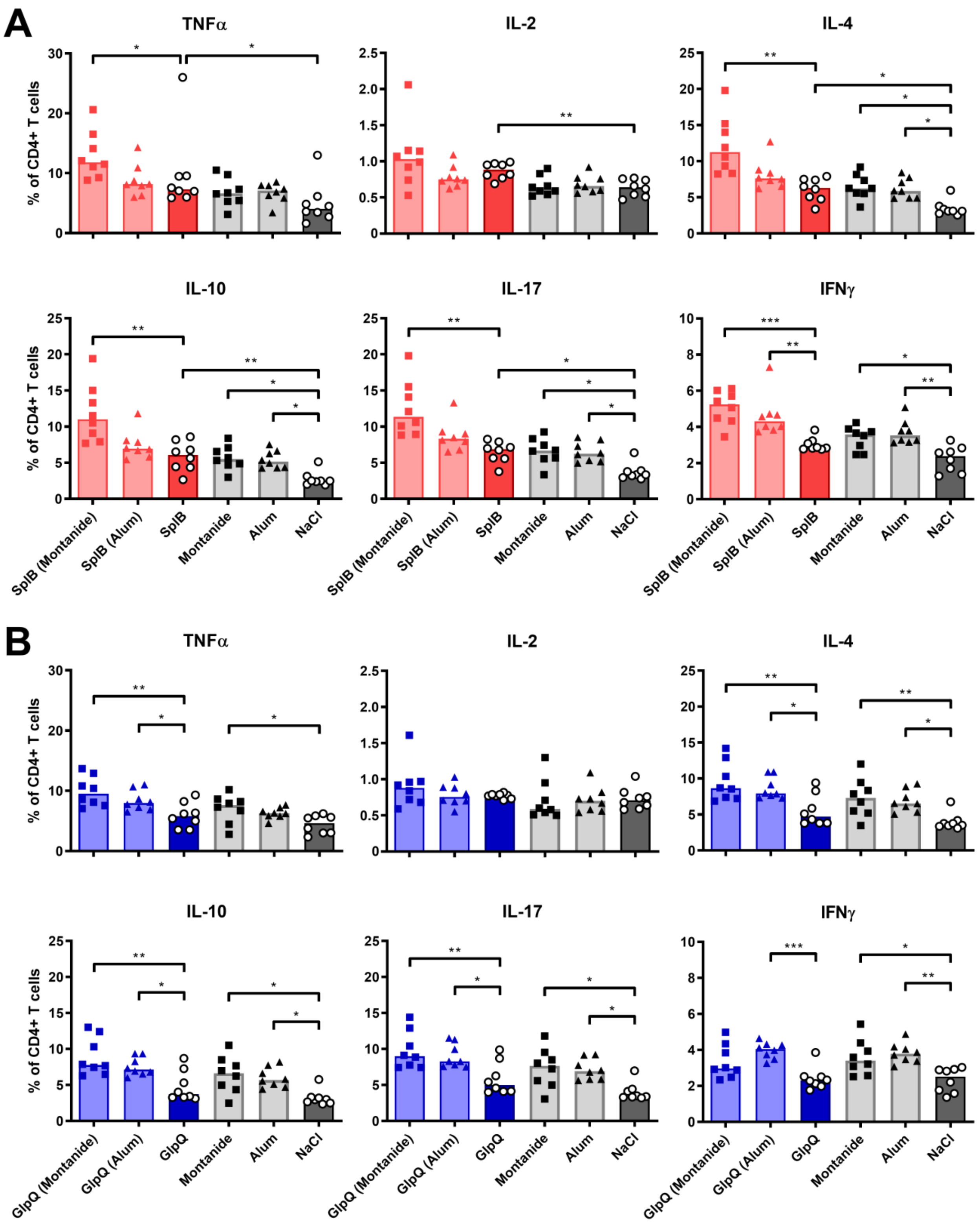
A
TNFα
IL-2
IL-4
IL-10
IL-17
IFNγ
% of CD4+ T cells
SplB (Montanide)
SplB (Alum)
SplB
Montanide
Alum
NaCl
B
TNFα
IL-2
IL-4
IL-10
IL-17
IFNγ
% of CD4+ T cells
GlpQ (Montanide)
GlpQ (Alum)
GlpQ
Montanide
Alum
NaCl

Supplementary Figure 3. Montanide increased the number of cytokine-positive $CD4^+$ T cells. Isolated splenocytes were restimulated with the indicated vaccine antigen overnight and $CD4^+$ T cells stained for the intracellular expression of TNFα, IL-2, IL-4, IL-10, IL-17 and IFNγ. **(A)** Results for *ex vivo* stimulation with SplB. **(B)** Results for *ex vivo* stimulation with GlpQ. Data are presented as median. n = 8 animals per group. *$p < 0.05$; **$p < 0.01$; ***$p < 0.001$.
